# Supplementary material for: Iatrogenic Hallux Varus in a Patient with Rheumatoid Arthritis
Source: Healthcare (Basel). 2025 Jan 21;13(3):217. doi: 10.3390/healthcare13030217 (PMC11817015; doi:10.3390/healthcare13030217)
Supplement: Supplementary file 1 [file healthcare-13-00217-s001.zip › healthcare-3396764-supplementary.pdf]

Supplementary File S1: CARE Checklist of Information to Include When Writing a Case Report

---

Title

1. *Iatrogenic Hallux Varus in a Patient with Rheumatoid Arthritis: Case Report*

Referenced in lines 2-4 of the manuscript

Key Words

2. *Hallux varus; iatrogenic; rheumatoid arthritis; pain; surgery; case report*

Referenced in lines 29-30 of the manuscript

Abstract

3a. *This case report highlights the unique complications of hallux valgus surgery in rheumatoid arthritis patients and provides insight into resolving surgical iatrogenesis.*
3b. *The patient presented with inflammatory pain and severe functional limitations due to hallux varus.*
3c. *The primary diagnosis was iatrogenic hallux varus, managed through metatarsophalangeal joint fusion and resection arthroplasty.*
3d. *This case emphasizes the importance of tailored surgical planning to avoid iatrogenic complications in rheumatoid arthritis patients.*

Referenced in lines 13-28 of the manuscript

Introduction

4. *The introduction discusses hallux varus deformity as a rare complication of hallux valgus surgery, particularly in rheumatoid arthritis patients, highlighting its frequency and clinical impact.*

Referenced in lines 32-55 of the manuscript

Patient Information

5a. *The report includes de-identified patient-specific information, such as clinical and radiological evaluations.*
5b. *The patient's primary concern was achieving pain-free ambulation.*
5c. *Her medical history was assessed, revealing no systemic, cardiac, or neurological conditions. Rheumatoid arthritis was the underlying condition associated with the surgical complication.*
5d. *Relevant findings from the patient's initial surgical intervention were deduced through radiological analysis.*

Referenced in lines 58-76 of the manuscript

Clinical Findings

6. *The patient presented with hallux varus deformity, claw positioning of the lesser toes, and an inability to wear shoes comfortably. Movement of the hallux was painful and non-reducible.*

Referenced in lines 62-65 of the manuscript

Chronology

7. *The patient was followed over 24 months, with consistent evaluations of pain and quality of life. A timeline summarizing the care process is provided below.*

Time Point	Event
**Pre-Surgery**	Patient diagnosed with rheumatoid arthritis and hallux valgus deformity.
**Initial Surgery**	Surgical correction of hallux valgus and Tailor's bunion.
**Post-Surgery Outcome**	Hallux varus deformity develops as a surgical complication.
**Day 0 (Current Surgery)**	Arthrodesis of the first MTP joint and resection arthroplasty performed.
**Week 1-6**	Weekly follow-ups, partial weight-bearing with a surgical shoe.
**Week 3**	Removal of K-wires from the lesser toes.
**Week 8**	Removal of MTP K-wires; transition to weight-bearing with sports shoes.
**Week 12**	Full recovery: pain-free walking and return to daily activities.
**Month 24**	Asymptomatic non-union observed in MTP fusion. Patient maintains full functionality and engages in sports.

Referenced in lines 58-108 of the manuscript

Diagnosis and Evaluation

8a. *Clinical, radiological, and laboratory evaluations were performed.*
8b. *Despite the absence of direct details about the first surgery, radiographic analysis allowed a reconstruction of the surgical history.*
8c. *The final diagnosis was iatrogenic hallux varus.*
8d. *After 24 months, the prognosis was favorable, with the patient maintaining pain-free functionality.*

Referenced in lines 68-76 of the manuscript

Therapeutics and Intervention

9a. *Therapeutic interventions included metatarsophalangeal joint fusion and resection arthroplasty.*
9b. *The patient received one daily dose of low molecular weight heparin for thromboembolic prophylaxis and dexketoprofen for pain relief.*
9c. *No changes in therapeutic interventions were required, as the initial strategy was effective.*

Referenced in lines 77-95 of the manuscript

Follow-Up and Results

10a. *Postoperative outcomes included correct toe alignment, pain relief, and restored functionality.*
10b. *Radiological follow-up revealed an asymptomatic non-union of the first MTP joint.*
10c. *Therapeutic adherence was monitored during weekly follow-ups for 6 months and later through quarterly evaluations.*
10d. *The non-union was an unanticipated finding but remained asymptomatic, without impacting the patient’s quality of life.*

Referenced in lines 96-124 of the manuscript

Discussion

11a. *Strengths and limitations of the case are discussed, focusing on the challenges of managing rheumatoid arthritis-related foot deformities.*
11b. *The discussion integrates relevant medical literature, comparing this case to existing evidence.*
11c. *The conclusions justify the surgical interventions chosen and highlight alternative techniques for similar cases.*
11d. *The primary takeaway is the need for comprehensive preoperative planning to address the unique challenges of rheumatoid arthritis-related foot deformities.*

Referenced in lines 131-174 of the manuscript

Patient Perspective

12. *The patient reported significant improvement in quality of life post-surgery, with no pain during walking and the ability to engage in sports and daily activities. She expressed satisfaction with the procedure despite the discomfort of wearing postoperative footwear.*

Referenced in lines 108-114 of the manuscript

Informed Consent

13. *Informed consent was obtained and is available upon request.*

Referenced in lines 241-248 of the manuscript
